# Disposable electrocatalytic sensor for whole blood NADH monitoring

**DOI:** 10.1038/s41598-022-20995-x

**Published:** 2022-10-06

**Authors:** JuKyung Lee, Han Na Suh, Saeyoung Ahn, Hye Bin Park, Jeong Yoon Lee, Hyung Jin Kim, Sang Hee Kim

**Affiliations:** 1 Department of Medical IT Convergence, Kumoh National Institute of Technology, Gumi, Gyeongbuk 39177 Republic of Korea; 2 Korea Institute of Toxicology (KIT), Jeongeup, Jeollabuk-do 56212 Republic of Korea; 3 NDD, Inc., Gumi, Gyeongbuk 39253 Republic of Korea; 4 Digital Health Care Research Center, Gumi Electronics and Information Technology Research Institute (GERI), Gumi, Gyeongbuk 39253 Republic of Korea; 5 Department of Bioactive Material Science, Korea Zoonosis Research Institute, Jeonbuk National University, Iksan, Jeollabuk-do 54531 Republic of Korea

**Keywords:** Enzymes, Diseases, Health care, Materials science

## Abstract

Monitoring nicotinamide adenine dinucleotide (NADH) is important because NADH is involved in cellular redox reactions and cellular energy production. Currently, few biosensors quantify NADH in whole blood. However, they still have limitations due to several defects, including poor repeatability, long analysis time, and their requirement of extra sample pretreatment. In this study, we developed electrocatalytic sensors using screen-printed electrodes with a redox-active monolayer 4′-mercapto-N-phenylquinone diamine formed by a self-assembled monolayer of a 4-aminothiophenol (4-ATP). We exhibited their behavior as electrocatalysts toward the oxidation of NADH in whole blood. Finally, the electrocatalytic sensors maintained stability and exhibited 3.5 µM limit of detection, with 0.0076 ± 0.0006 µM/µA sensitivity in a mouse’s whole blood. As proof of concept, a polyhexamethylene guanidine phosphate–treated mouse model was used to induce inflammatory and fibrotic responses, and NADH level was measured for 45 days. This work demonstrates the potential of electrocatalytic sensors to analyze NADH in whole blood and to be developed for extensive applications.

## Introduction

Maintaining mitochondrial function is critical to preserving homeostasis in living cells. Mitochondria are the cornerstone of life-supporting metabolic processes, such as energy transduction and calcium signaling in biosynthetic pathways^1^. Nicotinamide adenine dinucleotide (NADH), nicotinamide dinucleotide phosphate (NADP), and flavin adenine dinucleotide are the most important coenzymes that exhibit a beneficial effect on the mitochondrial state because adenosine triphosphate (ATP) production depends on the redox state of these coenzymes^2^. Among them, NADH is the most well-known biomarker of a cell’s redox state. Koidl et al.^3^ reported that NADH deficiency causes an energy production problem and induces Parkinson’s disease due to the lack of ATP. The conventional method for determining NADH is an optical assay using absorbance^4^ or fluorescence^5–7^ and a colorimeter^8^. The methods above are robust and standardized analytical methods for determining NADH. They also require high maintenance costs and sample volumes and exhibit a low limit of detection (LOD)^9^. Electrochemical (EC) biosensors for NADH emerged as an alternative analytical tool to conventional methods. The EC biosensor exhibits several advantages, including convenience, short analysis time, and their requirement of a small sample volume while maintaining high sensitivity and selectivity. The sensors above use the oxidation/reduction reaction of the NADH/NAD^+^ couplet. Specifically, NADH is oxidized to NAD^+^ at − 0.560 V vs. saturated calomel electrode and − 0.315 V vs. normal hydrogen electrode (NHE) (pH 7.0, 25 °C). The redox state of NADH/NAD^+^ is detected using an EC transducer^10^. However, a limitation emerged, which is that a large potential is required to oxidize NADH. This destroys the electrode surface due to the fouling effect and decreases efficiency because NAD^+^ is easily formed on the electrode surface through a redox reaction at the beginning of development^10^. To solve these problems, recent studies have been performed using various sensing configurations with a single mediator, such as (1) a nanostructure and gold nanoparticle^11^, (2) a conductive polymer^12^, and (3) an electrocatalytic enzyme reaction induced through surface modification, issued to reduce the redox potential of NADH^13^. However, nanostructures and nanoparticles have high maintenance costs and require complex fabrication steps. In addition, enzymes such as dehydrogenase have disadvantages, for example, signal reduction from fouling agents and interference from chemicals in a sample matrix. Despite the significant advantages, such as the high sensitivity of each method, the development of a rapid, cheap, and high-selectivity method that maintains sensitivity is still important in the NADH EC sensor field.

In this work, a “double-step electrochemical functionalization” to overcome the problems associated with measuring body fluids was developed using a screen-printed electrode (SPE). As an electrocatalyst, 4′-mercapto-N-phenylquinone diamine (NPQD) was immobilized on the SPE to decrease the redox potential of the NADH/NAD^+^ couplet. The SPE is a promising analytical tool as a cost effective NADH biosensor platform^14^. We systematically investigated electrocatalytic NADH oxidation, and the results indicated that the electrode exhibits an LOD of 3.5 µM and a sensitivity of 0.0076 ± 0.0006 µM/µA in mouse serum. Additionally, we investigated the electrocatalytic reaction in mouse blood to prove that the proposed sensor maintained its sensitivity by lowering the interference signal. A mouse model of a polyhexamethylene guanidine phosphate (PHMG-p)-induced lung injury was used, and NADH was quantified and compared using a control model. To the best of the authors’ knowledge, this study is the first attempt to quantify NADH electrochemically ex vivo*,* suggesting that the sensor is a promising tool for NADH monitoring of mitochondrial function and offers exciting opportunities to treat various diseases.

## Results and discussion

### Surface modification of the SPE

**Figure 1:**
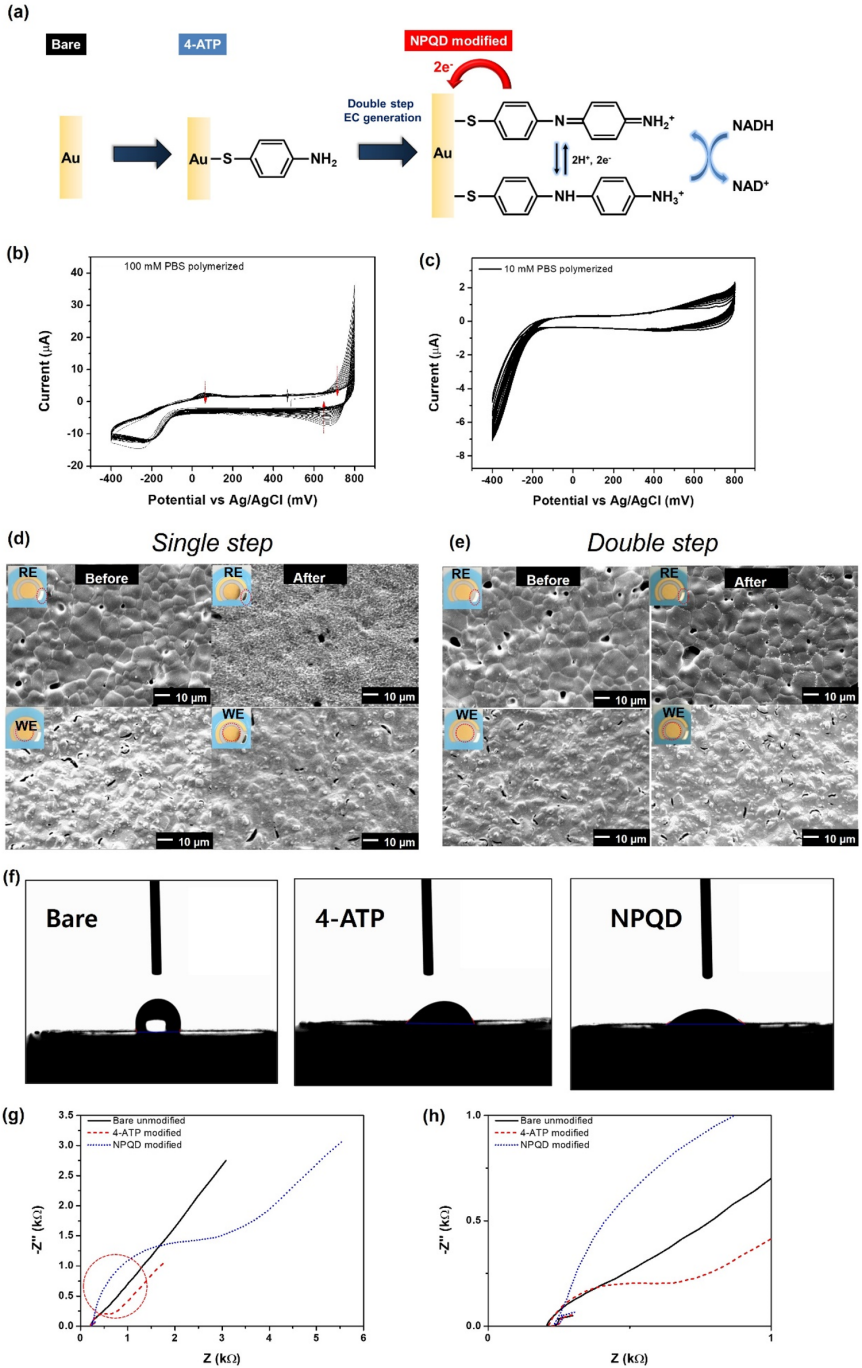
(**a**) Schematic diagram of NPQD modification. (**b**,**c**) CV data obtained during the electrochemical functionalization of the 4-ATP Au electrode in (**b**) 100 mM PBS (pH 7.4) and (**c**) 10 mM PBS (pH 7.4) after (**b**). The scan rate is 100 mV/s. (**d**,**e**) Single-step and double-step SEM images of the SPE surface after NADH measurement (five times) by changing the surface modification method. The reference and working electrodes are denoted by RE and WE. (**f**) Contact angle measurement of each electrode. (**g**) Nyquist plot for each electrode. EIS was performed in a 5 mM K_3_Fe(CN)_6_ + 0.1 M KCl solution. (**h**) The large graph is the expansion from the circled part of the Nyquist plot of (**g**).

Figure 1a shows a schematic illustration of the NPQD layer formation for the NADH electrocatalytic reaction. The aromatic diamine of NPQD is oxidized to diimine at the electrode. Subsequently, diimine, as an oxidant, attacks NADH in the solution. Additionally, it lowers the oxidation potential and enhances the current because diamines are electroactive, easily oxidized, and can transfer two electrons through NADH oxidation to NAD^+^. Surface modification was performed in two stages: modification of the SPE surface with 4-aminothiophenol (4-ATP), followed by EC functionalization of 4-ATP to generate the NPQD layer. In the first stage, a 4-ATP self-assembled monolayer (SAM) was prepared on the SPE by incubating the electrode in 10 mM 4-ATP dissolved in absolute ethanol for 2 h at room temperature. In the second stage, the generated NPQD substance was selected as the electrocatalyst and immobilized using electrochemical functionalization due to its good redox behavior and rigid structure^15^. Although NPQD functionalization is similar, we selected a different electrochemical functionalization method and used a different substrate as the SPE. Figure 1b shows repeated cyclic voltammogram (CV) data obtained during the functionalization of 4-ATP in 100 mM phosphate buffer saline (PBS) with a pH of 7.2). The large anodic and cathodic peaks near 0.7 V and the small cathodic peak at 50 mV smoothly decreased using a CV cycle. This result is differs slightly from previous reports because the cathodic current at 0.55 V and the anodic current at − 0.2 V decreased. The reversible redox peak at 0.23 V emerged through a repeating CV cycle. However, the redox peak at 0.23 V was not observed, and we speculated that this was due to differences in our proposed system. The SPE comprises gold (Au) working, Au counter, and silver (Ag) reference electrodes; 4-ATP was immobilized on the working and counter electrodes. This caused an electrochemical motion different from that in previous reports because only the Au working electrode, not the counter electrode, was functionalized. We observed that the CV signal was unstable even when we increased the cycle number to 100. Additionally, the electrode was damaged during NADH sensing. The stability of the EC signal is important for generating the NPQD layer. Therefore, we modified the electrochemical functionalization method to maintain stability. To prove this assumption, we performed electrochemical functionalization using a low concentration of PBS buffer (10 mM con, pH 7.4), after using a high concentration of PBS buffer, for stabilization while maintaining other CV parameters. We called this method is “double-step functionalization” to distinguish previous result as “single-step” because single-step functionalization only used 100 mM PBS concentration. Although the current, approximately − 0.4 V, did not exhibit significant fluctuation, the CV signal was stable, and changes observed in the 100 mM PBS were not observed in the 10 mM PBS buffer (Fig. 1b,c).

The NADH-sensing performance of the single-step electrochemical functionalization using 100 mM PBS buffer and the double-step electrochemical functionalization sequentially using 100 mM and 10 mM PBS buffers are shown in the scanning electron microscopy (SEM) images (Fig. 1d,e). A stability problem arose when repeated measurements were performed with a single step. Following five measurements, as shown by SEM, the reference region of the single-step-modified electrode was burned, and the grain boundary dimmed. Given the problem with the reference electrode, the working region was also damaged because it could not maintain a constant potential. However, concerning repeated measurements, the stability of the double-step-modified electrode was significantly improved compared to that of the single-step-modified electrode. Moreover, the grain boundaries of Ag and Au were observed.

NPQD functionalization was also demonstrated by the contact angle measurement of a water droplet since the –NH functional group in NPQD is polarized and hydrophilic. The contact angle is 108.4° for the bare electrode and 54.4° for the 4-ATP-modified electrode, whose value is not so different from that of the bare electrode (Fig. 1f). However, the NPQD-modified electrode showed a much lower value of 33.9° due to the amine group of NPQD. In addition to surface angle measurement, the generation of NPQD as a redox-active monolayer was also observed using electrical impedance spectroscopy (EIS). Figure 1g,h show the Nyquist plots of the impedance for NPQD monolayer formation. EIS was used to investigate unmodified, 4-ATP-, and NPQD-modified Au surfaces at a constant redox specie concentration of Fe(CN)_6_^3−/4−^. We found a charge transfer resistance (R_ct_) expressed by the diameter of a semicircle in the Nyquist plot of the NPQD-modified surface, which had a higher value (3133 Ω) than the unmodified and 4-ATP-modified electrodes (433 Ω and 819 Ω, respectively). *Consequently, a rigid layer was generated from the benzene dimer structure, and these SAMs have a longer carbon length than the 4-ATP monolayer generated from monobenzene. That is a rigid layer generated from dibenzene of NPQD creates a denser and thicker electrical isolation layer. Thus, the charge transfer resistance (R*_*ct*_*) is higher in the NPQD layer than in the 4-ATP layer.* Additionally, a 45° line in the Nyquist plot indicates a Warburg region of semi-infinite diffusion of a electron species in the modified electrode, and the NPQD-modified electrode shows a diffusion process governed by mass transport of redox molecules from the solution to the electrode^16^. Table 1 shows the specific EIS parameters of each electrode. The electric circuit was characterized based on the Randles circuit.

**Table 1:** Electrical circuit simulation by a Nyquist plot.

	Parameter	Bare	4-ATP	NPQD
Fitting parameter	R_sol_ (Ω)	11.68	12.11	12.34
	R_ct_ (Ω)	433	808	3145
	W	5.86e−4	192.5	208.2
	CPE-T	1.67e−5	9.33e−4	9.75e−4
	CPE-P	0.785	2.14e−5	4.52e−5
Circuit				

R_ct_: charge transfer resistance, R_sol_: solution (bulk) resistance, W: Warburg coefficient, CPE-P: capacitance phase element P value, CPE-T: constant phase element T value.

### NADH sensor sensitivity and selectivity

**Figure 2:**
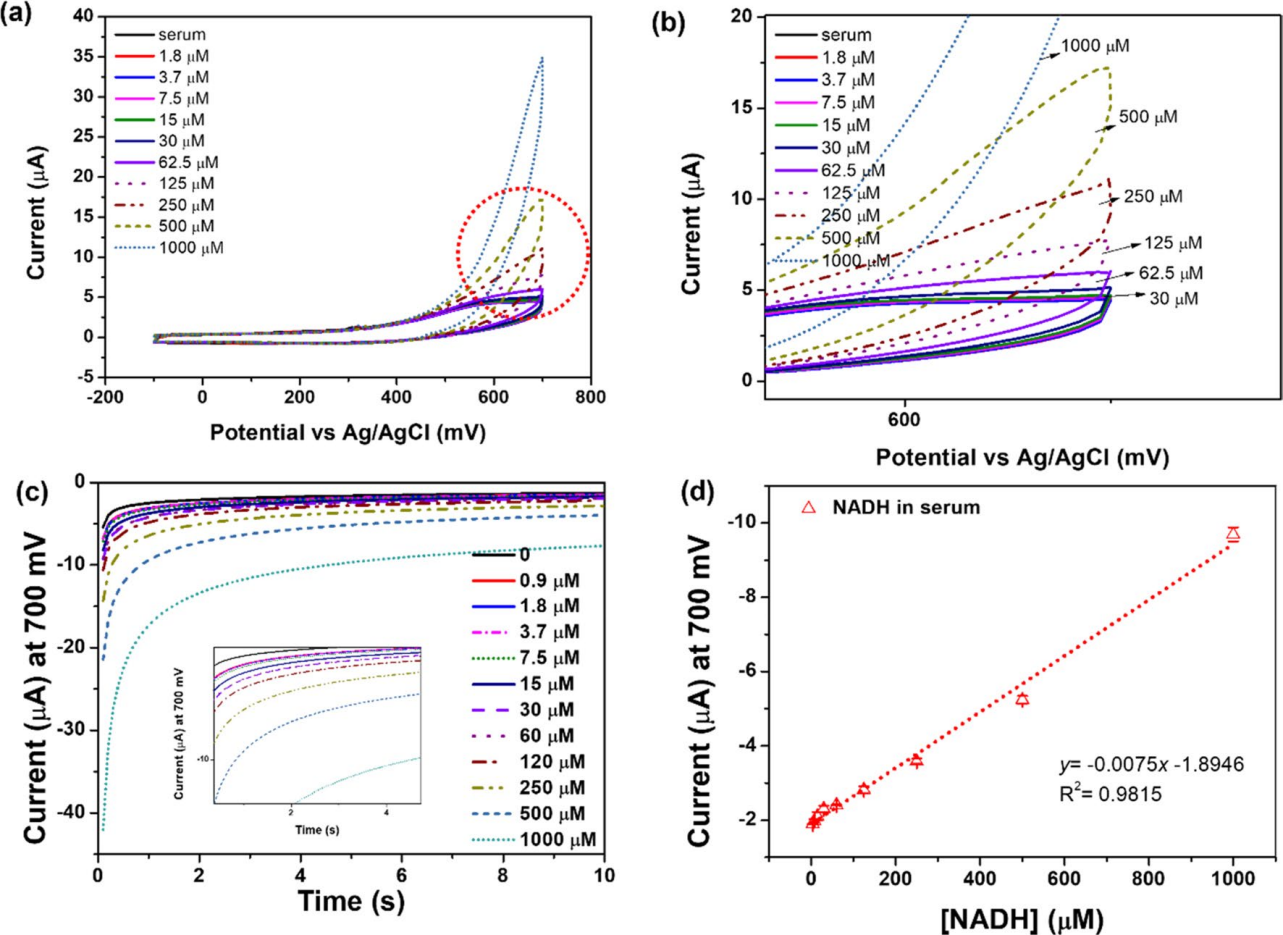
(**a**–**d**) Electrochemical results for NADH in the mouse serum. (**a**) Voltammogram results (potential range: from − 100 to 700 mV). (**b**) CV from the circled part of graph (**a**). (**c**) NADH quantification measured by CA. (**d**) Calibration plot of NADH in the mouse serum (0–1000 µM) (n = 5). The current at 10 s in the CA graph was used for NADH quantification.

The electrocatalytic activity of the NPQD-Au electrode for NADH oxidation in the cell culture medium has already been shown^17^. The electrocatalytic sensor was used to detect NADH in different electrolytes using a cell culture medium because monitoring the NADH concentration in the cell is essential in directly investigating the mitochondrial dysfunction status^18–20^. The CV signal in the mouse serum is similar to the signal in the medium, proving that the proposed sensor also works in the mouse serum (Fig. 2a,b). The current was caused by NADH oxidation to NAD^+^, which regenerates the diamine, as shown in the schematic diagram (Fig. 1a). To oxidize NADH to NAD^+^, a potential is required as the driving force. The current remains unchanged with an increasing NADH concentration from − 100 to 400 mV. However, the current increases with increasing NADH concentration from 500 to 700 mV. We fixed the potential at 700 mV because the current is considerably changed with changing NADH concentrations. Chronoamperometry (CA) was used to make a calibration plot of NADH because a short and fixed potential analysis time (10 s) per sample was required when compared with CV. The current was stable at 10 s; thus, we gathered the current at 10 s with changing NADH concentrations (Fig. 2c).

Finally, the NADH concentration in the mouse serum followed the adjusted equation I (nA) = {(− 7.55)·[NADH], µM − 1.89} (R^2^ = 0.981), with a range of linearity between approximately 16–1000 µM and an LOD of 3.5 µM (Fig. 2d). The sensitivity of the developed sensor was improved using double-step electrofunctionalization when compared with the single-step functionalization performed by Raj et al. (LOD: 3.5 µM vs. 10 µM)^15^. Additionally, the stability was also improved even for repeated measurements. Given the NPQD functionalization in the electric double layer, we can assume that the difference is due to the NPQD generation efficiency, related to the length of the electric double layer. The Debye length (i.e., the thickness of the double layer that forms at the charged surface in 100 mM PBS buffer) is 0.34 nm, although it is approximately 0.7 nm in the 10 mM PBS buffer^21,22^. A thicker double layer leads to deeper penetration of the target NPQD’s electric potential and exceeds the Debye length in the 100 mM PBS buffer. However, the penetration rate is lower in the 10 mM PBS buffer, which displays a greater Debye length. The NPQD generation rate increases because the penetration rate of the electric potential on the electrode decreases. This contributes to the sensitivity because NADH is easily oxidized by transferring electrons from NPQD.

**Figure 3:**
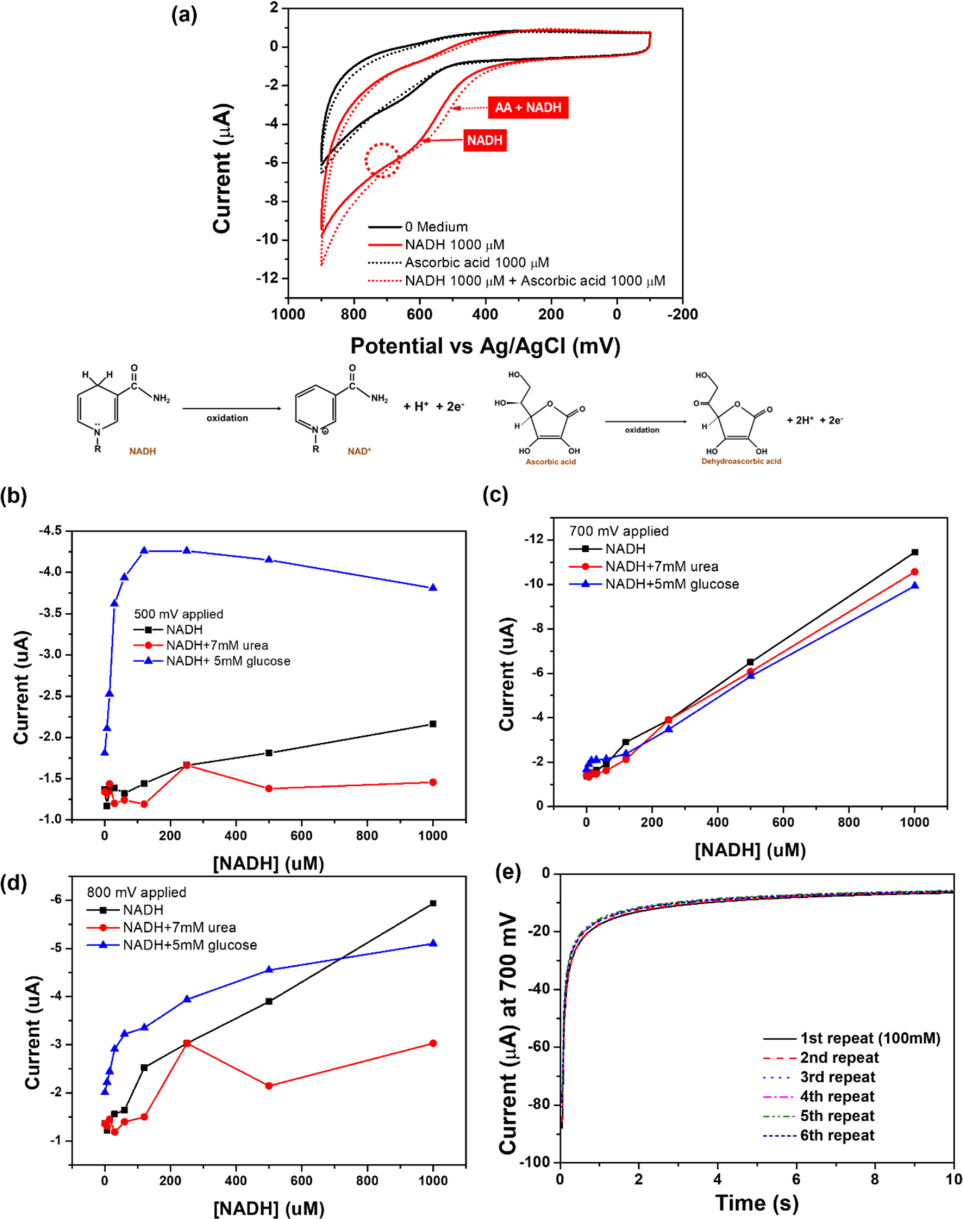
(**a**) Selectivity test with NADH and AA. (**b**–**d**) Signal difference for NADH with the addition of other impurities at (**b**) 500 mV, (**c**) 700 mV, and (**d**) 800 mV. (**e**) Stability test of NADH with repeated measurement (500 μM NADH solution was used).

The main problems with the NADH sensor are instability due to fouling on the electrode surface when NADH is oxidized to NAD^+^ and the selectivity due to NADH oxidation, which is largely influenced by the oxidation of ascorbic acid (AA)^23–27^. A selectivity test was performed by using the same concentrations of NADH and AA (1000 µM), and no significant difference was found when 1000 µM AA was added (the dotted black line), compared with a medium containing no AA and NADH (the solid black line). The detection of NADH with our sensor was not affected by AA, as evidenced by an NADH signal (solid red line) from a 600 mV to 700 mV range similar to the signal of an NADH + AA complex (dotted red line) (Fig. 3a). AA and NADH were measured in a cell culture medium to demonstrate the selectivity of the measurement. Different concentrations of other biomarkers were added to the electrode surface to capture NADH, a biomarker for lung disease. This is because the cell culture medium contains many other impurities, such as albumin, glucose, and other inorganic salts, that may interfere with obtaining an accurate signal for NADH. Typically, glucose and urea exist at high concentrations in the biological samples; thus, observing selectivity using this marker is adequate. The concentrations of glucose and urea were fixed at 5 mM and 7 mM, which are the normal levels. The current was not stabilized at the 500 mV and 800 mV potentials (Fig. 3b–d). Furthermore, we observed that the CV graph was not stabilized at the 500 mV and 800 mV potentials. However, the signal, which is similar even in the presence of glucose and urea, was stabilized at 700 mV, meaning that only NADH was oxidized at 700 mV potential, demonstrating the excellent selectivity of this modified electrode.

Repeated measurements were performed in the presence of a high concentration of NADH (500 µM) to demonstrate stability. Surprisingly, the signal for the 5^th^ run had a similar value to the signal for the 1st run (0.17% difference), which demonstrates that our NADH sensor maintained its electrocatalytic ability after several measurements were made even under harsh conditions, such as in the presence of a higher concentration of NADH (Fig. 3e). The achieved sensitivity and sensing range were applied to cell studies and nonclinical animal experiments (e.g., mice) because the known concentrations in animal cells were approximately 0.3 mM^28–32^.

### Electrochemical monitoring of mitochondrial NADH from an ex vivo system

To apply the proposed technique in an ex vivo system, we investigated changes in the NADH concentrations in whole blood over time during the initiation and development of PHMG-p-induced lung inflammation and fibrosis. Specifically, NADH is an important component of redox systems that plays a key role in various human diseases, including cancer and lung disorders. NADH concentration can change during many pathophysiological processes. We hypothesized that NADH could correspond to a potential biomarker reflecting the aforementioned pathophysiological processes. Additionally, a change in the NADH level can precede the onset of a metabolic disorder, as evidenced by the previous result, which indicated a relationship between cell viability and the magnitude of the change in the amount of NADH. To test the hypothesis, NADH sensing was performed using mouse whole blood to determine whether its performance was comparable regarding sensitivity and selectivity maintenance. First, the variables involved in preparing the NADH electrocatalytic sensor in the blood were optimized. Second, each sample’s variable was individually tested using a single NPQD-modified SPE, which included (a) the optimization of the ethylene-diamine-tetraacetic acid (EDTA) concentration used as an anticoagulant, (b) blood dilution factor, and (c) applied potential. The details of the optimization studies are summarized in Table 2.

**Table 2:** Optimization of the experimental variables affecting the performance of the NADH electrocatalytic sensor using mouse blood.

Variable	Tested	Selected
Blood dilution factor	1:10, 1:20, 1:40, 1:80, 1:160	1:40
EDTA concentration, mM	0, 0.05, 0.5, 5, 50, 500	5
Applied potential, mV	500, 600, 700, 800	700

Matrix effect coefficient:

1 $$\left(\mathrm{\%}\right)Matrix \; effect=\left[ \frac{Response \; \left( in \; spiked \; sample\right)}{Response \; \left(in \; standard \; solution\right)}-1\right]\times 100$$

Matrix coefficient of the serum:

2 $$\left(\mathrm{\%}\right)Matrix \; effect \;\left(serum\right)=\left[\frac{0.00958}{0.01012}-1\right]\times 100=2.34\mathrm{ \%}$$

Matrix coefficient of blood:

3 $$\left(\mathrm{\%}\right)Matrix \; effect \; \left(blood\right)=\left[\frac{0.00865}{0.01012}-1\right]\times 100=4.53\mathrm{ \%}$$

**Figure 4:**
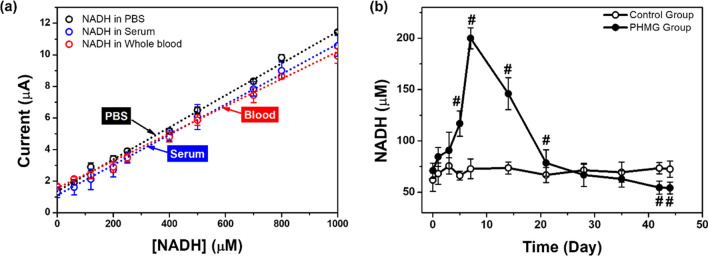
(**a**) Calibration plot for NADH obtained with pure PBS buffer, mouse serum, and blood (n = 5). (**b**) NADH profiles of the control and PHMG-p exposure groups obtained using an electrocatalytic assay (n = 5).

There was no significant effect on the NADH electrocatalytic sensor response with the presence of any substances in the serum or blood, demonstrating that excellent selectivity and sensitivity were maintained (Fig. 4). Finally, the matrix coefficient obtained using Eqs. (1–3) was 2.34% for serum and 4.53% for blood. Following the optimization, we examined the effect of potential interfering compounds and the NADH in various biological samples on the electrochemical responses obtained using the NPQD-modified SPE. Matrix effect experiments were performed to determine problems with the sensor using spiked samples, and a known amount of NADH was added to the PBS buffer, mouse serum, or mouse blood.

After optimizing and constructing a calibration plot, we performed time-course analyses of NADH on the blood obtained from the PHMG-p-treated mice using the proposed electrocatalytic sensor. Although PHMG-p exhibits biocidal properties relative to various bacteria, yeast, and molds, epidemiological studies revealed that inhalation of PHMG-p induced lung and physiological toxicity in Korea^33,34^. Previously, using histologic analyses of hematoxylin and eosin and Masson’s trichrome stained preparations^35^, we established an animal model of PHMG-p-induced lung inflammatory and fibrotic responses and reported that PHMG-p induced polymorphonuclear cells (PMN) and macrophage-dominant lung inflammation in Week 1 and marked lung fibrosis from Weeks 2 to 10.

**Figure 5:**
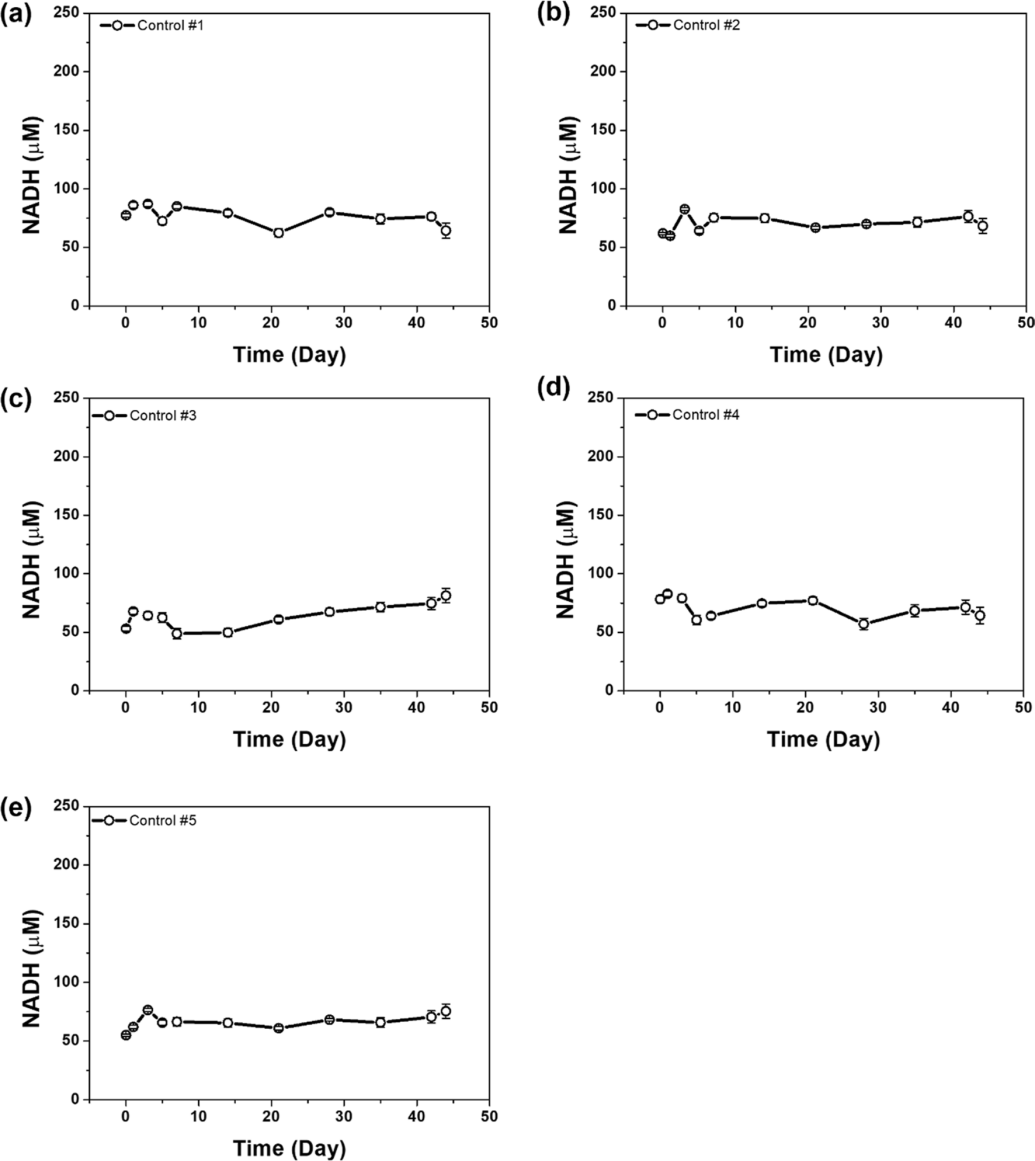
NADH profiles of the control groups as obtained using an electrocatalytic assay (n = 5).

**Figure 6:**
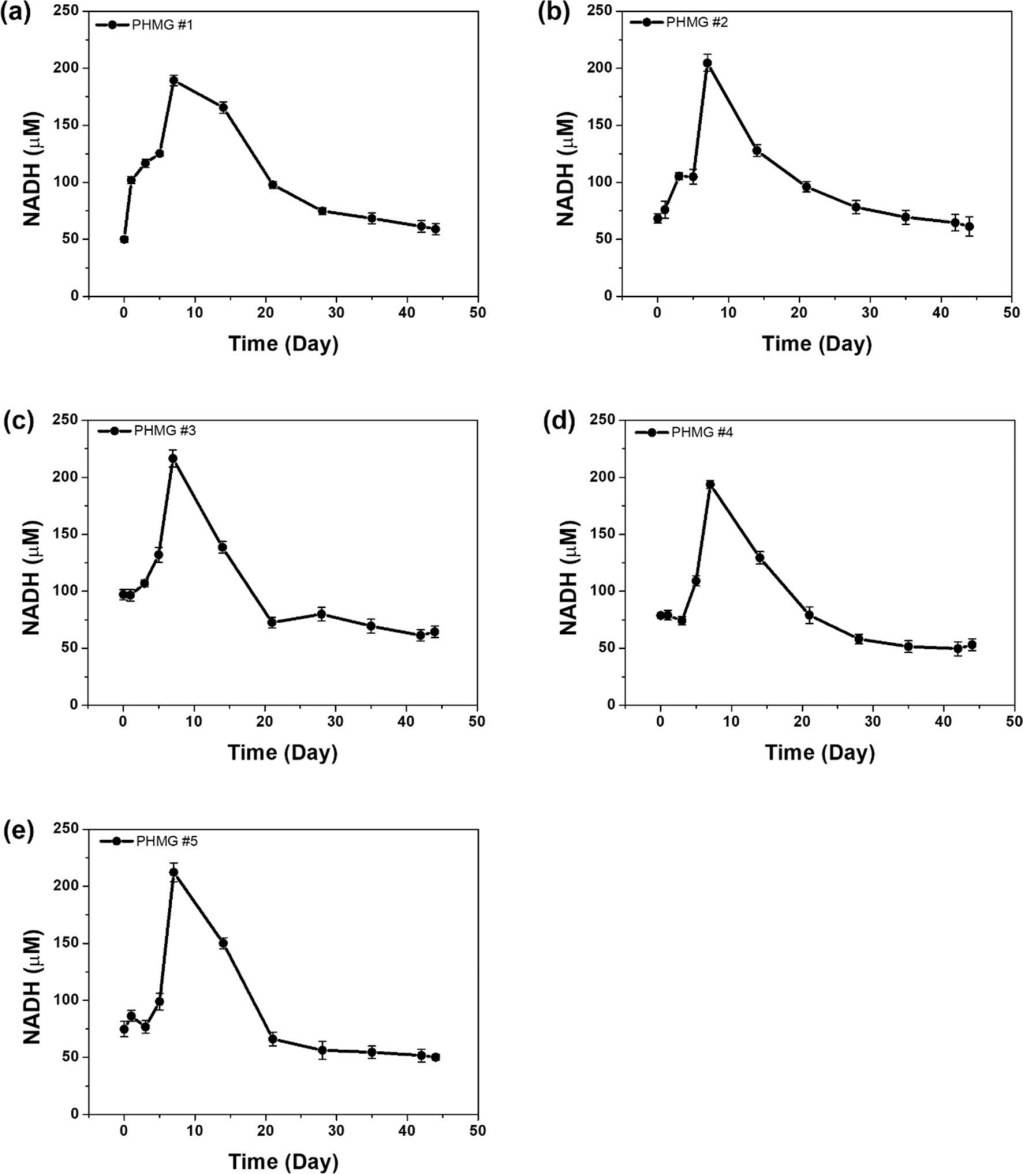
NADH profiles of the PHMG-p exposure groups obtained using an electrocatalytic assay (n = 5).

On Day 7, the NADH concentration was shown to peak in the blood of PHMG-p-treated mice compared to the control mice and subsequently decreased continuously until Day 44 (Fig. 4b). On Day 44, the NADH concentration in the control mice was 72.55 ± 7.99 µM and that in the PHMG-p-treated group was 54.24 ± 5.77 µM. Our results indicated that changes in the NADH concentration were related to lung inflammatory and fibrotic responses caused by PHMG-p. Individual data are shown in Figs. 5 and 6. The results also suggested that our surface-modified SPE-based electrocatalytic system can experimentally monitor the ex vivo release of NADH.

Continuous monitoring of NADH in ex vivo studies is one application of our sensor. The results suggest that the technique enables monitoring of mitochondrial energetics by measuring the change in NADH concentration during the initiation and development of PHMG-p-induced lung inflammation and fibrosis (or lung injury).

Table 3 shows the recent electrochemical analysis of NADH and its specifications. As shown the table, nanostructure and nanoparticles (Ag, Au) were used to quantify NADH because AuNPs function as an efficient mediator. Moreover, GCE and graphene based on the carbon electrode with surface modification of a conductive polymer were also used to quantify NADH and observed very low LOD. Enzymes, such as alcohol dehydrogenase and glutamic dehydrogenase, were used for NADH quantification. Many research groups have developed biosensors, but only a few groups achieved the result of NADH in urine, saliva, and sweat as body fluids, which have a complex solution. Our electrocatalytic sensor is beneficial because it comprises one simple step, consumes a low sample volume (50 μL), and has a low LOD. Moreover, this is the first attempt to use it as a tool to observe the relationship of NADH in mouse blood with toxicological effect induced by PHMG. As previously mentioned, NADH is an important marker of the cellular redox state and mitochondrial function and is crucial in maintaining cellular homeostasis by regulating cellular metabolism and energy production^40^. Nevertheless, NADH accumulates when the cellular redox state is out of balance due to various physiological stimuli (e.g., exercise, diet, or hormones) and stressors, which leads to various human diseases, including lung diseases caused by mitochondrial dysfunction. Our results indicated that in the PHMG-p group, NADH rapidly increased during initial lung injury and then significantly decreased during a prolonged lung injury with fibrotic features. The results were consistent with the NADH pattern displayed due to a rapid imbalance between NADH generation and oxidation in an ischemia–reperfusion animal model^38^. Although further examining the relationship between NADH and various diseases, including lung diseases, is essential, the results indicate that the sensor can be applied to an ex vivo system.

**Table 3:** Comparison of NADH detection based on an electrochemical reaction.

Electrode	LOD	Linear range	Sample	Ref
NPQD-Au/single step	10 µM	10–190 µM	PBS	^15^
AuNPs/PB/GCE	0.21 µmol/L	0.5–1000 µmol/L	PBS	^36^
PEDOT CM/GCE	5.3 µM	20–240 µM	PBS	^37^
MoSe_2_/HEG	1 µM	1–2380 µM	PBS	^38^
POA-Ag/GCE	0.05 µM	5–270 µM	Urine, PBS	^39^
NPQD-Au/double-step/SPE	3.5 µM	16–1000	PBS, serum, blood	This work

PEDOT: poly(ethylene dioxythiophene), CM: colloidal microparticles, GCE: glassy carbon electrode, HEG: hydrogen-exfoliated graphene, ADH: alcohol dehydrogenase, POA: poly(o-anisidine), NPQD: 4′-mercapto-N-phenylquinone diamine, SPE: screen-printed electrode.

## Conclusion

We demonstrated the development and application of a double-step functionalization by lowering the electrolyte concentration from 100 to 10 mM in a PBS buffer to prepare an electrocatalytic-modified electrode for NADH oxidation and biosensing. The NADH sensor exhibits good analytical performance, with a linear response of 16 and 1000 µM and an LOD of 3.5 µM in the mouse serum. Additionally, the sensor permits the sensing of NADH redox signaling without the interference of impurities while maintaining high sensitivity and selectivity, making it an appropriate platform for continuous monitoring of NADH. The ex vivo PHMG-p-treated mice blood analysis substantiated the performance of the NADH sensor. Future efforts should aim to establish an NADH mechanism and its effect on PHMG-p-induced lung disease. Such efforts should lead to a better understanding of PHMG-p cytotoxicity.

## Methods

### Reagents and chemicals

PHMG-p was generously donated by SK chemicals (Seongnam, South Korea). K_3_Fe(CN)_6_, 4-ATP, 100 mM and 10 mM of Dulbecco’s phosphate-buffered saline (DPBS), Tween 20, absolute ethanol, EDTA, and mouse serum were purchased from Sigma-Aldrich (St. Louis, USA) and used without further purification. All reagents used in the investigation were of analytical grade.

### Apparatus and electrode

Commercial SPE (Model no: DRP 220 AT, Φ = 4 mm) was purchased from Metrohm DropSens (Asturias, Spain). CA, and CV, EIS were performed with a potentiostat obtained from CH Instruments (Texas, USA; Model no: CH 660C). All electrochemical measurements were performed at room temperature in a Faraday cage to ensure electromagnetic shielding.

### Surface modification of the SPE

Before assembly, the SPE was pretreated by placing a 50 µL drop of a 10 mM H_2_SO_4_ solution on it, and cyclic voltammetry was performed from 0 to 1.8 V at a scan rate of 100 mV/s to remove dust. Subsequently, the SPE was washed with deionized (DI) water and dried with nitrogen. After it was dried in a stream of N_2_, the 4-ATP SAM was prepared on the SPE by incubating the electrode in 10 mM 4-ATP dissolved in absolute ethanol for 2 h at room temperature. Subsequently, the SPE was washed with absolute ethanol for 1 min and washed again with 0.05% Tween 20 in 10 mM DPBS (pH 7.2) to remove the remaining chemicals. An NPQD layer was generated on the Au electrode through a two-step electrochemical surface modification. After drying in a stream of N_2_, 50 µL of 100 mM DPBS buffer (pH 7.2) was dropped on the SPE, and CV was performed by applying a potential range between 0.8 and − 0.4 V 30 times. The electrode was washed using 10 mM DPBS buffer (pH 7.2), and a CV step identical to the preceding step was performed by changing the 100 mM DPBS buffer to 10 mM DPBS buffer. Following surface modification, the electrode was washed with 0.05% Tween 20 in 10 mM and kept in a 10 mM DPBS buffer.

### Characterization of the surface-modified electrode

#### Scanning electron microscopy

The SEM micrographs were recorded with an EVO MA 10 (Carl Zeiss Ag, Germany). The samples were sputtered with Au before microscopic analyses.

#### Contact angle measurements

The hydrophilic properties of the surface-modified layer were characterized using contact angle measurement. The contact angle data of each layer was obtained by dropping 10 µL of DI water on the modified surface of the Au working electrode, and the image was analyzed using the ImageJ contact angle program. All measurements were performed on the air-facing surfaces of the films. Five different measurements were performed on each modified electrode, and the standard error was determined.

#### Electrochemical impedance spectroscopy measurement

The impedance spectra were recorded from 1 MHz to 0.1 Hz at an AC signal amplitude of 50 mV. A CV with a different scan rate and impedance was recorded in 5 mM K_3_Fe(CN)_6_ + 0.1 M KCl in a pH 7.4 PBS buffer as the electrolyte solution at room temperature.

#### Calibration of NADH in mouse serum

To construct a calibration curve for NADH, first, various concentrations of NADH (0–1000 µM) were separately prepared in a 1:10 diluted mouse serum. Subsequently, CV at a scan rate of 100 mV/s was used to electrochemically characterize the electron transfer between the NPQD-modified surface and NADH under both conditions after dropping 50 µL of an NADH solution on the electrode.

The CA was recorded by applying a fixed potential determined by CV. A computer-controlled EC analyzer was used for signal readout at a fixed potential of 0.7 V (culture medium) with a pulse width corresponding to 10 s and a sample interval corresponding to 0.05 s. By plotting the current intensities when a steady state was reached, a standard curve for NADH was obtained. Several calibration curves were obtained (n = 5) with each concentration under the same conditions to calculate the average and standard deviation. The LOD was determined by gathering the background signal and three standard deviations (3σ).

### Ex vivo evaluation

#### Ethics statement

All the experiments were approved by the Institutional Animal Care and Use Committee of the Korea Institute of Toxicology (KIT) and conducted based on the guidelines of the Association for Assessment and Accreditation of Laboratory Animal Care International (B218004). In addition, the present study was carried out following the ARRIVE guidelines.

#### Animals

Six-week-old male C57BL/6 mice were purchased from Orient Bio, Inc. (Seongnam, Korea). The mice were housed in an environmentally controlled animal room maintained at a temperature of 22 ± 3 °C, relative humidity of 50 ± 20%, and an air ventilation rate of 10–20 changes/h, with a 12 h light/dark cycle. Sterile pelleted food for experimental animals (PM Nutrition International, Richmond, USA) and UV-irradiated (Steritron SX-1; Daeyoung, Inc., Korea) and filtered (1 μm) tap water was provided. The mice were acclimatized for 6 d. All experiments were approved by the Institutional Animal Care and Use Committee of the KIT and conducted based on the guidelines of the Association for Assessment and Accreditation of Laboratory Animal Care International (B218004).

### Treatments and experimental groups

Mice (n = 10) were randomly assigned to one of the following twoweight-matched experimental groups (six mice per group) using the Pristina System (Version 7.3; Xybion Medical System Co., USA): mice treated with saline for Group 1 (control) and mice treated with PHMG-p for Group 2. The mice in Group 1 were instilled with 50 μL of saline solution using an automatic video instiller. Mice in the PHMG-p (25%; SK Chemicals, Seoul, Korea) groups received a single intratracheal instillation (ITI) of 1.1 mg/kg PHMG-p in 50 μL saline solutions through the same route.

### Intratracheal instillation

A mouse was anesthetized with isoflurane and placed on its back in a head-up position on a flat board at an inclined angle. Concerning the ITI, the upper incisors of the mouse were held by a rubber band fixed on the board. The tongue was gently moved to one side of the oral cavity, and then the mouse was instilled with 50 µL of each sample using an automated syringe pump and video instiller.

### Blood collection

Before commencing collection, we diluted a 5 mM EDTA solution with PBS. A mouse was gently placed in a restrainer, and its tail was washed and cleaned using a wipe with 70% alcohol. After grasping the distal end of the tail vein line, we inserted a 1 mL needle with the bevel side up and parallel to the skin. The needle was placed correctly in the vein and was slowly removed afterward. Blood was collected from the vein until at least 10 μL of blood was obtained. A 10 μL aliquot of blood was directly mixed with a 90 μL EDTA solution in a tube and stored at − 80 °C.

### Quantification of NADH in mouse blood

To quantify NADH in mouse blood, 50 µL of diluted blood extracted as detailed in the previous section was dropped on the NPQD-modified electrode. NADH quantification was performed within 10 min after extraction to prevent contamination. Five replicates were used for each sample, and non-PHMG-p-treated mice were used as the positive control group. A potential of 0.7 V was applied, and the current was read at a stabilization state after 20 s.

### Statistical analysis

All values were expressed as the mean ± standard deviation. Statistical analyses were performed using a two-tailed t-test, with statistical significance defined as *p* < 0.05. All assays were run five times, and the mean and standard deviation were calculated at each concentration to generate the calibration curve. Each replicate was measured with a new SPE. The analyte NADH was made at each time measurement to maintain the fresh condition. A nonlinear curve fitting was performed with the Origin 8.0 program.

## Data Availability

All data generated or analyzed during this study are included in this published article.
