# Author Correction: Disposable electrocatalytic sensor for whole blood NADH monitoring

**DOI:** 10.1038/s41598-022-26355-z

**Published:** 2022-12-19

**Authors:** JuKyung Lee, Han Na Suh, Saeyoung Ahn, Hye Bin Park, JeongYoon Lee, Hyung Jin Kim, Sang Hee Kim

**Affiliations:** 1 Department of Medical IT Convergence, Kumoh National Institute of Technology, Gumi, Gyeongbuk 39177 Republic of Korea; 2 Korea Institute of Toxicology (KIT), Jeongeup, Jeollabuk-do 56212 Republic of Korea; 3 NDD, Inc., Gumi, Gyeongbuk 39253 Republic of Korea; 4 Digital Health Care Research Center, Gumi Electronics and Information Technology Research Institute (GERI), Gumi, Gyeongbuk 39253 Republic of Korea; 5 Department of Bioactive Material Science, Korea Zoonosis Research Institute, Jeonbuk National University, Iksan, Jeollabuk-do 54531 Republic of Korea

Correction to: *Scientific Reports* https://doi.org/10.1038/s41598-022-20995-x, published online 06 October 2022

The Acknowledgements section in the original version of this Article was incomplete.

“This research was supported by the Kumoh National Institute of Technology (2019-104-044) and the Hustar Medical Industry Innovation University Project and Nano-Material Technology Development Program through the National Research Foundation of Korea (NRF) funded by the Ministry of Science and ICT (NRF2021M3H4A407927511 and 2021M3H4A4079264).”

now reads:

“This research was supported by the Kumoh National Institute of Technology (2019-104-044) and the Hustar Medical Industry Innovation University Project, S3310771 (small and medium business administration) and Nano-Material Technology Development Program through the National Research Foundation of Korea (NRF) funded by the Ministry of Science and ICT (NRF2021M3H4A407927511 and 2021M3H4A4079264) and Nanomedical Devices Development Project of NNFC in 2022."

The original Article has been corrected.

